# Knowledge and use of antibiotics in Thailand: A 2017 national household survey

**DOI:** 10.1371/journal.pone.0220990

**Published:** 2019-08-09

**Authors:** Sunicha Chanvatik, Hathairat Kosiyaporn, Angkana Lekagul, Wanwisa Kaewkhankhaeng, Vuthiphan Vongmongkol, Apichart Thunyahan, Viroj Tangcharoensathien

**Affiliations:** 1 International Health Policy Program, Ministry of Public Health, Nonthaburi, Thailand; 2 National Statistical Office, Ministry of Information and Communication Technology, Bangkok, Thailand; University of Campania, ITALY

## Abstract

**Background:**

The Thailand National Strategic Plan on Antimicrobial Resistance (AMR) 2017–2021, endorsed by the Thai Cabinet in 2016, aims to increase public knowledge about antibiotics and AMR awareness by 20% by 2021. This study assesses the prevalence of antibiotics use, clinical indications and sources; knowledge and access to information related to antibiotics and AMR; and factors related to level of knowledge and access to information among Thai adult population.

**Methods:**

An AMR module was developed and embedded into the 2017 Health and Welfare Survey; a cross-sectional, two-stage stratified sampling, nationally representative household survey carried out biannually by National Statistical Office. The survey applied a structured interview questionnaire. The survey was conducted in March 2017 where 27,762 Thai adults were interviewed of the AMR module. Data were analyzed using descriptive and inferential statistics.

**Results:**

The one-month prevalence of antibiotic use was 7.9% for three common conditions; flu (27.0%), fever (19.2%) and sore throat (16.8%). The majority of antibiotics (70.3%) were provided by public or private healthcare facilities, and 26.7% by pharmacies. Thai adults have low levels of knowledge about antibiotics; only 2.6 gave correct answers to all six statements related to antibiotics, while 13.5% gave wrong answers to all six statements. A few factors associated with knowledge and having received information on antibiotics were assessed. People who have higher education levels, and belong to richer wealth quintiles, and receive antibiotics and AMR information have significantly higher levels of knowledge about antibiotics. In the last 12 months, only 17.8% of respondents had heard information about the proper use of antibiotics and AMR; mostly from doctors (36.1%), health workers (24.8%) and pharmacists (17.7%).

**Conclusions:**

There is a large gap of public knowledge about the use of antibiotics. The main communication channel is through healthcare professionals, which indicates they are key persons in communicating information about the proper use of antibiotics to the public.

## Introduction

In order to improve awareness and understandings about antimicrobial resistance (AMR) as recommended by the World Health Organization (WHO) [1], countries need to develop a sustainable system for monitoring the population’s knowledge about antibiotics and awareness of AMR in order to inform effective interventions. In 2015, WHO conducted a multi-country public awareness survey in twelve countries, two from each of the six WHO regions. In the South-East Asia Region, India and Indonesia were two sample countries [2]. A series of special Euro-barometers 338, 445, 478 and Flash Eurobarometer had been conducted in European countries [3–6]. Other high-income countries have also generated evidence about knowledge and awareness of antibiotic use among the general population [7–9].

The Thailand National Strategic Plan on AMR 2017–2021 (NSP-AMR) was endorsed by the cabinet in August 2016 [10]. One of the five goals is to increase public knowledge of antibiotics and awareness on AMR by 20% by 2021. In 2017, the National Statistical Office (NSO) and the International Health Policy Program (IHPP) of the Ministry of Public Health, Thailand jointly developed a module to assess the use of antibiotics, levels of knowledge about antibiotics and sources of information on the appropriate use of antibiotics and AMR for the first time among the Thai population. In order to sustain the monitoring of knowledge about antibiotics in the Thai population, the AMR module was integrated into the Health and Welfare Survey (HWS), an existing health survey established by the NSO since 1974.

Due to lack of knowledge among lay people, lack of access to qualified and affordable health services, self-medication of antibiotics is a widespread phenomenon worldwide [11]; which can result in drug interactions and the emergency of AMR. The prevalence of antibiotic self-medication ranged from 26–100% in Bangladesh, and 73–82% in Sudan [11]. Self-medication with antibiotics is a complex phenomenon which is driven by a variety of determinants [12], from the patients, healthcare professional and system levels. Countries in South East Asia Region had particularly a comparatively high level of inappropriate use of self-medicated antibiotics [13].

Recognizing the complexity of antibiotics self-medication, government should develop effective multifaceted interventions that target healthcare professionals and patients simultaneously; while in parallel, ensure provision of adequate and affordable access to health care services can prevent self-medication with antibiotics.

In response to the gap of understanding about the use of antibiotics in the population; this study aimed to generate baseline evidence on the one-month prevalence of antibiotic use, clinical indications and sources of antibiotics; the levels of knowledge about antibiotics and AMR among the Thai adult population, and assessed factors associated with knowledge and having received public information on proper use of antibiotics. This evidence is essential to assess progress in implementing the NSP-AMR.

## Materials and methods

### Development of AMR module

The AMR module was modified from the “Antimicrobial Resistance Eurobarometer Survey” with additional questions on knowledge of antibiotics specifically designed to suit the national context.

The AMR module had three sections. The first section asked about the use of antibiotics in the last month, the sources of antibiotics and the reasons for using them. The second section asked about knowledge of antibiotics, which was assessed using true, false statements and one question. To ensure accuracy of Yes and No answer, a “do not know” answer also provided. Section three explored whether respondents had received information during the last twelve months about antibiotics and AMR, the sources of such information and whether the information changed their view and future practice of using antibiotics. See Table 1.

**Table 1:** AMR module embedded in 2017 HWS.

		Contents	Choices of answer
**I. USE OF ANTIBIOTICS**			
AB1		Have you taken any antibiotics orally such as tablets, powder or syrup in the last month?	Yes/ No/ Don’t know
AB2 (IF ‘YES’ to AB1)		Where did you obtain the last course of antibiotics that you used?	Choices of answer: Health center/ Community hospital/ General or regional hospital/ University hospital/ Other public hospital/ Private hospital/ Private clinic/ Pharmacy/ Grocery store/ Some left over from the previous treatment (your own and others)/ Mobile medical Unit/ Others (Specify)
AB3 (IF ‘YES’ to AB1)		What was the reason for last taking the antibiotics that you used? (Multiple answers possible)	Choices of answer: Pneumonia, Bronchitis, Rhinitis and rhinopharyngitis throat, Flu/ Influenza, Sore throat, Cough, Fever, Headache, Diarrhea, Urinary tract infection, Skin or wound infection, Others (Specify), Don’t know
**II. KNOWLEDGE ABOUT ANTIBIOTICS**			
AB4_1	Please tell me whether you think it is true or false. “Antibiotics kill viruses” (The correct answer is a false statement.)		True, False, Don’t know
AB4_2	Please tell me whether you think it is true or false. “Antibiotics are effective against colds and flu” (The correct answer is a false statement.)		True, False, Don’t know
AB4_3	Please tell me whether you think it is true or false. “Unnecessary use of antibiotics makes them become ineffective” (The correct answer is a true statement.)		True, False, Don’t know
AB4_4	Please tell me whether you think it is true or false. “Taking antibiotics often has side-effects such as diarrhea” (The correct answer is a true statement.)		True, False, Don’t know
AB4_5	Please tell me whether you think it is true or false. “Antibiotics are not equal to anti-inflammatory drugs” (The correct answer is a true statement.)		True, False, Don’t know
AB5	When do you think you should stop taking antibiotics once you have begun a course of treatment?		Choices of answer: When your illness is better, When you get full course of antibiotics (from doctor's or health professionals recommendation), Others (Specify), Don’t know
**III. PUBLIC INFORMATION ABOUT THE PROPER USE OF ANTIBIOTICS AND AMR**			
AB6		In the last 12 months, do you remember getting any information about not taking antibiotics unnecessarily, for example for a cold or the flu, or information on antimicrobial resistance?	Yes/ No/ Don’t know
AB7 (IF ‘YES’ to AB6)		Whom did you get this information about not taking antibiotics unnecessarily?	Choices of answer: A doctor told me, A pharmacist told me, Another health professional (e.g. nurse, physical therapist) told me, A family member / Friends told me, I saw it on a TV advertisement, I saw it on the internet / social media, I saw it on a leaflet/poster, I read it in a newspaper, I saw it on the TV news, I heard it on the radio, Others (Specify), Don’t know
AB8 (IF ‘YES’ to AB6)		Did the information that you received change your views on using antibiotics?	Yes/ No/ Don’t know
AB9 (IF ‘YES’ to AB6)		On the basis of the information you received, how do you now plan to use antibiotics? (Multiple answers possible)	Choices of answer: When you think you need an antibiotic, You will no longer self-medicate with antibiotics, You will no longer take antibiotics without a prescription from a doctor, You will no longer keep left over antibiotics for the next time you are ill, Others (Specify), None, Don’t know

The content validity was assessed by five experts from different fields (pharmacologists, public health specialists and health promotion specialists) on the logic and clarity of the content. The pilot testing was conducted with a sample of 40 individuals for improving the quality of questions. These 40 individuals were randomly selected from general staffs in the Ministry of Public Health (representing lay people); with an aim to improve the content validity. We found the contents are easily understood and accurate with no controversy. There is minor amendment of the questionnaire after piloting.

### The Health and Welfare Survey

The HWS, established by the NSO since 1974, is a national representative cross-sectional household interview survey carried out biannually by the NSO. The survey was conducted in March 2017.

The HWS applied a stratified two-stage sampling. Greater Bangkok and the remaining provinces constituted strata, with 77 strata altogether. Each stratum was divided into municipal (urban) and non-municipal (rural) areas. In the first stage, sampling enumeration areas (EAs) from urban and rural area were selected using probability proportional to size based on total household numbers. In the second stage, private households were the sampling units. In each sampled EA, a systematic random sample of private households was selected.

The HWS contains comprehensive sets of questions categorized into modules, including 1) socio-economic and demographic parameters; 2) types of insurance coverage; 3) illness and use of services: 3.1—illness in the last month, use and choices of services and out-of-pocket payments by households; 3.2—use of prevention and health promotion services in the last 12 months; 3.3—admission in the last 12 months, uses, choices and payment; 3.4—use of dental services in the last 12 months; 4) unmet healthcare needs, 5) food consumption; 6) AMR module; and 7) housing characteristics and ownership of durables for computing wealth index and quintiles.

The objective of the sample design for HWS 2017 was to produce statistically reliable estimates for each indicator, represented at national level, urban and rural areas, and five geographical regions. The sample size is not design to represent provincial level.

Stratified two-stage sampling approach was used. The first stratum is all 77 provinces (including Bangkok); the second stratum in each province has two sub-strata, namely urban and rural areas. Enumeration areas (EA) for urban and rural were calculated based on proportional probability to size of population. Total 1,990 sample EAs were selected from the total national 127,460 EA. From the sampling frame in each of the selected EA, 16 and 8 households were systematically randomly selected from urban and rural EAs. This resulted in total 27,960 sample households for HWS.

Of these 27,960 samples households, only 23,411 households had responded, with a response rate of 83.7%. The remaining are emptied houses or cannot be identified due to error of addresses. In these 23,411 households, there were a total of 65,781 members of all ages who participated in the HWS. Of the total 65,781 household members, only 27,762 who are adult members, age 15 years or above who presented on the survey time and date, had completed the face-to-face interview of AMR module. The interview was conducted in Thai and took about 60 minutes. Data were collected using a software program.

Prior to the survey, all NSO interviewers across 77 provinces were trained through video conference on the AMR manual produced by the researchers. The contents include background, how antibiotics are different from other medicines, global and national concern of AMR, issues around self-medicated antibiotics, clinical conditions, potential sources of antibiotics distribution and the contents of true and false statements. The goal of training is to ensure high quality, reliable and valid information from the survey.

On definition of urban and rural areas, NSO adheres to Ministry of Interior’s definition; that urban area means municipality according to the Municipality Act, B.E. 2496 (1953). Urban area also includes special local government established by its own legislation such as Bangkok Metropolitan and Pattaya City. Areas outside municipality are rural areas.

Prior to interview, NSO interviewers provided survey background and objective and assured confidentiality to respondents. According to Section 15 of The Statistics Act, B.E. 2550 (2007), personal information obtained under this Act shall be strictly kept confidential. A person who performs duty or a person who has the duty of maintaining such information cannot disclose it to anyone who does not have a duty according to this Act. No consent is required by Section 9 of the Act, for surveys conducted by national statistical authority. Further, participants can withdraw from the interview at any time they so wish.

### Data analysis

Data were analyzed using STATA/IC (version 14.2). Descriptive measures were presented in percentages. Differences in distribution between groups were compared using logistic regression with an estimate of 95% confidence intervals (CIs). For all tests, p-values of 0.05 or less were used to determine the level of significant difference. Multivariate analysis was employed to assess the relationship between levels of knowledge about antibiotics and receiving information and demographic data such as gender, age, area of residence, education level and wealth status as shown in Table 2. The variables were selected by reviewing literatures and analysing the significant bivariate association. We used the cut off point for the outcome variable of level of knowledge as lower and equal to and higher than three correct answers (>50% of total questions).

**Table 2:** Multivariate analysis of demographical factors associated with high level of knowledge and receiving information about antibiotic use and AMR.

Characteristic	Knowledge about antibiotics				Public information on use of antibiotics and AMR			
	Number of respondents with > 3 correct answers (%)	Adjusted OR	95% CI	p-value	Number of respondents who received information (%)	Adjusted OR	95% CI	p-value
**Gender**				0.574 (LR test)				<0.001* (LR test)
Male	2,568 (23.0)	Reference			1,888 (37.0)	Reference		
Female	3,834 (23.1)	0.98	0.93–1.04	0.574	3,222 (63.0)	1.18	1.11–1.26	<0.001*
**Age group, years**				<0.001* (LR test)				0.018* (LR test)
15–24	430 (26.1)	Reference			273 (5.3)	Reference		
25–59	4,398 (25.4)	0.99	0.88–1.12	0.914	3,275 (64.1)	1.13	0.98–1.30	0.099
> or = 60	1,574 (17.9)	0.82	0.72–0.94	0.005*	1,562 (30.6)	1.22	1.05–1.42	<0.001*
**Area of residence**				0.218 (LR test)				0.049 (LR test)
Urban	3,824 (24.9)	Reference			2,898 (56.7)	Reference		
Rural	2,578 (20.8)	0.96	0.91–1.02	0.218	2,212 (43.3)	1.07	1.00–1.14	0.05
**Education level**				<0.001* (LR test)				<0.001* (LR test)
Uneducated	191 (12.8)	Reference			139 (2.7)	Reference		
Primary school	2,847 (18.1)	1.24	1.06–1.46	0.009*	2,672 (52.3)	1.82	1.52–2.18	<0.001*
Secondary school	2,150 (28.1)	1.82	1.54–2.15	<0.001*	1,511 (29.6)	2.08	1.72–2.52	<0.001*
University and above	1,208 (43.7)	3.00	2.50–3.60	<0.001*	783 (15.3)	2.82	2.29–3.46	<0.001*
**Wealth quintile**				<0.001* (LR test)				<0.001* (LR test)
Q1	1,063 (15.8)	Reference			882 (17.3)	Reference		
Q2	1,156 (19.9)	1.20	1.10–1.32	<0.001*	883 (17.3)	1.13	1.02–1.25	0.022*
Q3	1,242 (21.9)	1.25	1.13–1.37	<0.001*	1,041 (20.4)	1.38	1.25–1.52	<0.001*
Q4	1,438 (26.6)	1.40	1.27–1.54	<0.001*	1,238 (24.2)	1.74	1.58–1.92	<0.001*
Q5	1,503 (36.3)	1.75	1.58–1.94	<0.001*	1,066 (20.8)	1.83	1.64–2.05	<0.001*
**Receiving information**				<0.001* (LR test)				
No	4,196 (22.6)	Reference						
Not sure	317 (7.8)	0.31	0.28–0.35	<0.001*				
Yes	1,889 (37.0)	1.84	1.72–1.97	<0.001*				

*refer to statistically significance

Likelihood Ratio Test (LR test) evaluate the difference nested models which one model restricts a parameter to zero by removing the predictor variables from the model.

## Results

### Prevalence of antibiotic use, sources and clinical indications

The prevalence of antibiotic use in the preceding month among the adult population was 7.9% of the total 27,762 adults individual who were successfully interviewed of the AMR module, while 12.3% of respondents could not confirm whether the drugs they used in the previous month were antibiotics or not. Among the 2,024 individuals who reported antibiotics use, 50.3% received it from public health facilities, 20.0% from private health facilities, 26.7% from retail pharmacies and 3.0% from grocery stores. Both retail pharmacies and grocery stores were categorized as self-medication of antibiotics; hence the prevalence of self-medicated antibiotics was 29.7% in Thai adult population.

**Fig 1:**
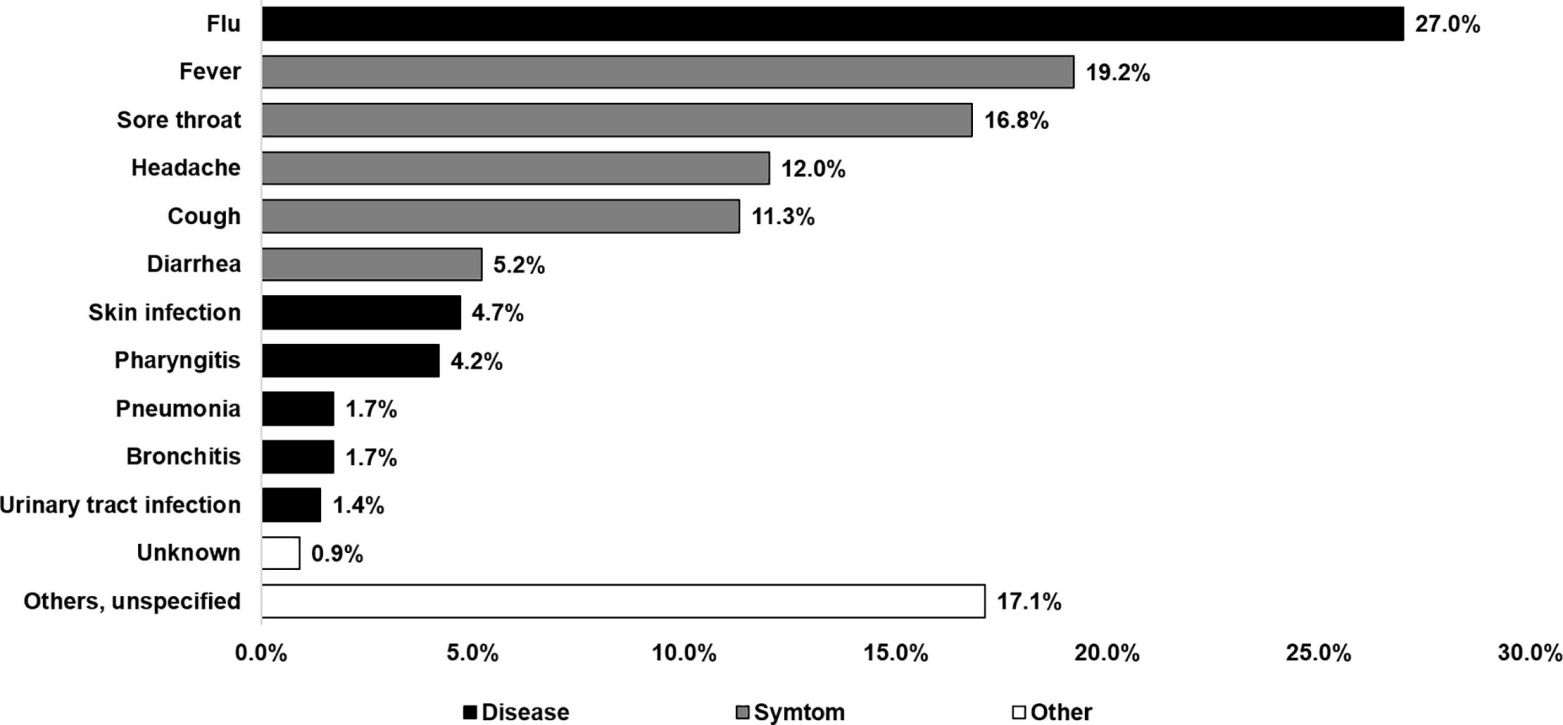
Indication of antibiotic use. Note that total percentages were more than 100% due to multiple answers.

In Fig 1 we classified self-reported clinical indications for antibiotic use into three groups. Most antibiotics (64.5%) were used to treat symptoms (fever 19.2%, sore throat 16.8%, headache 12%, cough 11.3% and diarrhea 5.2%). Antibiotics were also reported for treatment of illnesses, such as flu (27%), skin infection (4.7%) and pharyngitis (4.2%). Interestingly, 17.1% of population answered that they had other symptoms and diseases which were unspecified. However, 17.4% of them responded more than one answer which were mostly symptoms and flu.

### Knowledge about antibiotic

**Fig 2:**
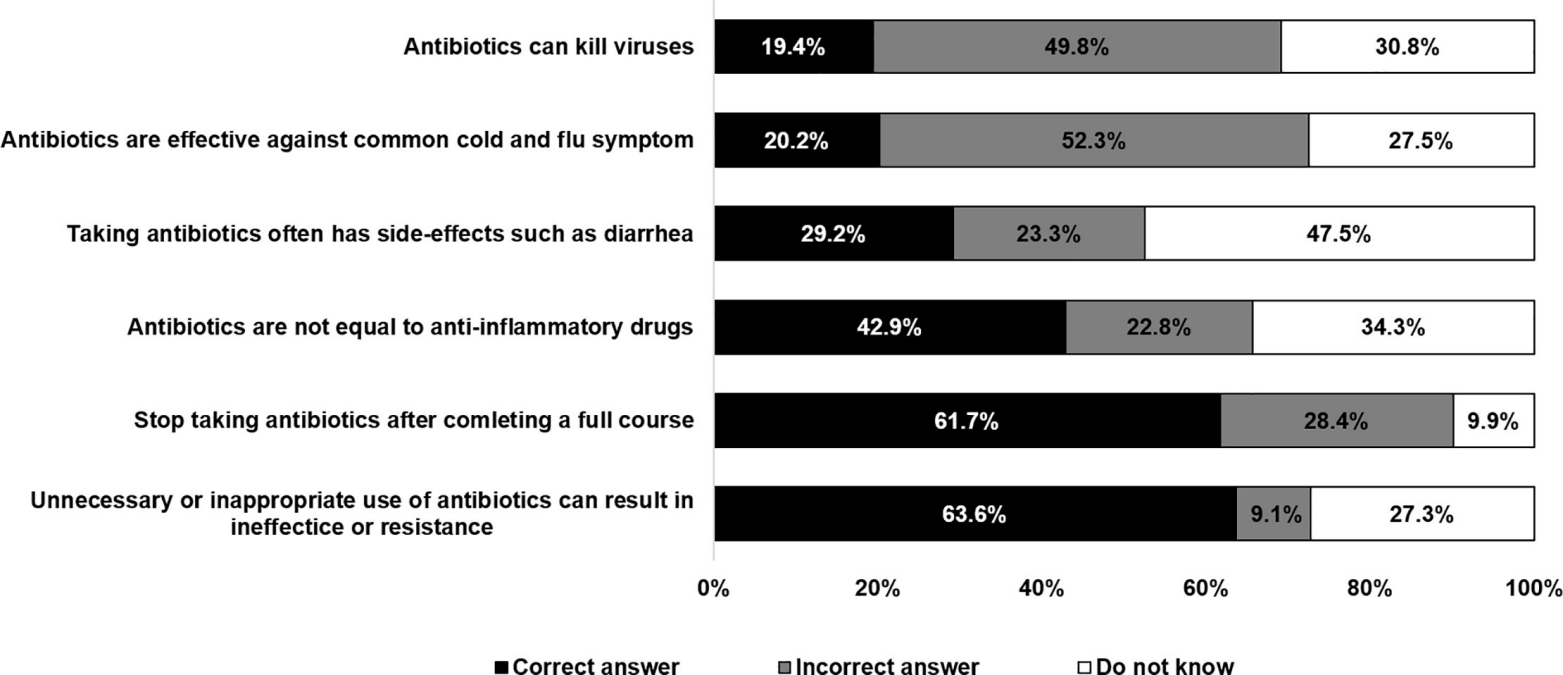
Respondents’ knowledge on antibiotics, HWS 2017.

The majority of respondents (63.6%) correctly recognized that unnecessary or inappropriate use of antibiotics can result in ineffective treatment or resistance; 61.7% agreed that they should stop antibiotics after completing a full course of treatment; 42.9% gave the correct answer that antibiotics are not anti-inflammatory drugs. However, a half of respondents (52.3%) gave the wrong answer to the statement that antibiotics can cure common cold and flu symptoms; and 49.8% of respondents wrongly thought that antibiotics can kill viruses. Almost half of respondents (47.4%) did not know that excessive use of antibiotics can result in side effects such as diarrhea. See Fig 2.

**Fig 3:**
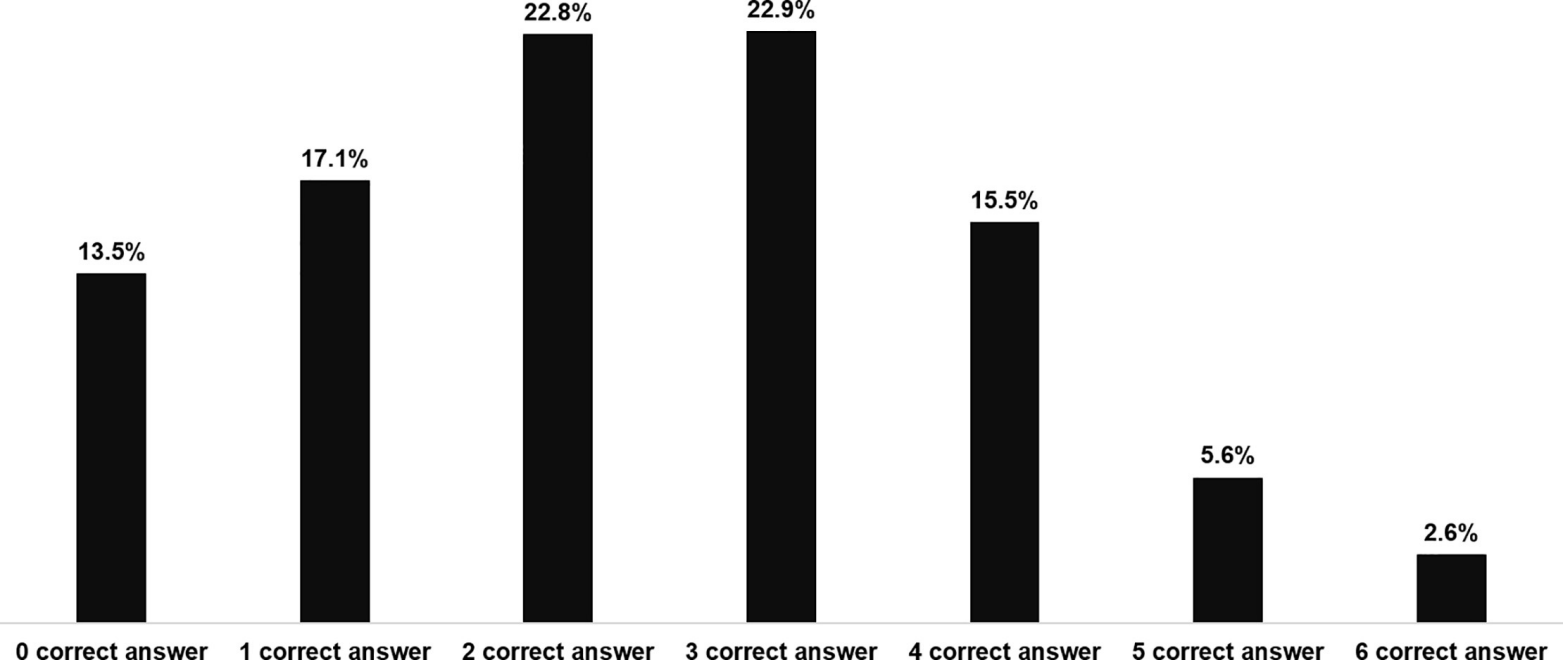
Percentages of respondents who gave correct answers.

Only 2.6% of all adult respondents gave correct answers to all six statements; 8.2% of respondents gave five or more correct answers; less than a quarter of respondents (23.7%) gave four or more correct answers; less than a half of respondents (46.6%) gave three or more correct answers. Alarmingly, 13.5% of Thai adults gave wrong answers to all six statements (see Fig 3).

The bi-variate analysis showed that age, area of residence, educational level and wealth quintile were significantly associated with level of knowledge. However, previous study showed that age and receiving information also influenced the knowledge about antibiotics [14, 15]. The significant variables from literatures and bivariate analysis were assessed as inputs in multivariate analysis. The multivariate analysis in logistic regression showed that a respondent’s higher education, richer wealth quintile and receiving information on antibiotic use and AMR have significant effects on knowledge about antibiotics. Specifically, respondents who had a bachelor degree or higher were three-fold more likely to have better knowledge than those who were uneducated (OR = 3.00; 95%CI = 2.50–3.60, p-value<0.001). Accordingly, respondents who belonged to the richest wealth quintile had a 1.8-fold higher knowledge than the poorest quintile (OR = 1.75; 95%CI = 1.58–1.94, p-value<0.001). Respondents who had received to public information about antibiotics and AMR were 1.8-fold more likely to have higher knowledge than those who had not received to information (OR = 1.84; 95%CI = 1.72–1.97, p-value<0.001).

On the right panel of Table 2, logistic regression shows that females, older age groups, those with higher education level and in richer wealth quintiles have statistically significant higher probability of receiving public information about the appropriate use of antibiotics and AMR. For example, in terms of receiving information on antibiotics and AMR, females have 1.18 times higher changes than males (OR = 1.18; 95%CI = 1.11–1.26, p-value<0.001) and respondents older than 60 years have 1.22 times higher chances than the 15–24 age group. Respondents who had a bachelor degree or higher are 2.8 times more likely to receive information than those who were uneducated. Respondents who belonged to the richest wealth quintile have a 1.8 times higher chance of receiving information than the poorest quintile.

### Public information on appropriate use of antibiotics and AMR

**Fig 4:**
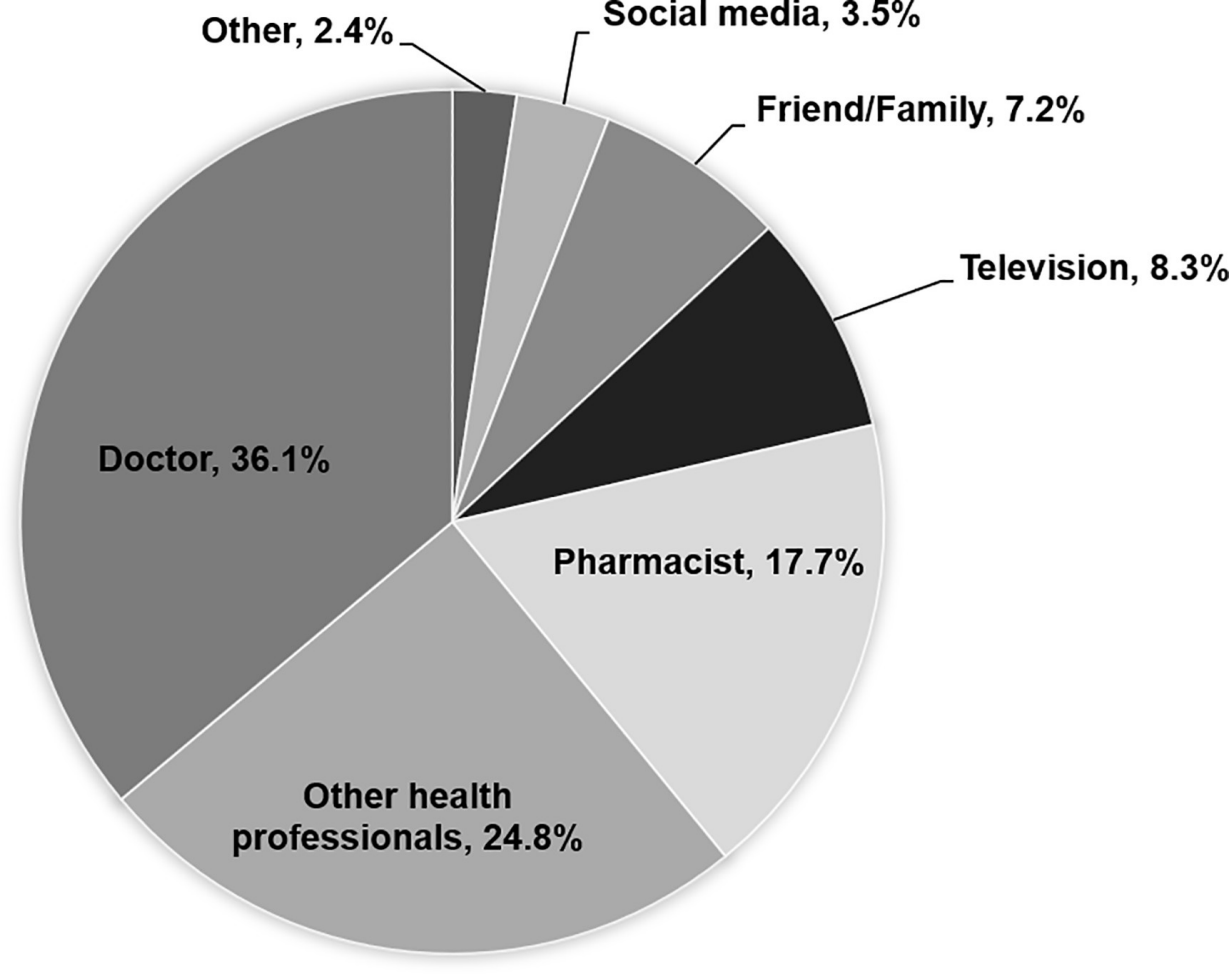
Source of information on antibiotics and AMR in the last year. Note: Others include leaflets, posters, newspapers and radio broadcasting.

Only 17.8% of respondents had received information about the appropriate use of antibiotics and AMR in last 12 months. Three common sources of information were doctors (36.1%), health workers (24.8%) and pharmacists (17.7%). Other sources such as conventional media (television and social media) played a minor role contributing 8.3% and 3.5% respectively, while 7.2% of respondents received information from family and friends (see Fig 4).

## Discussion

This is the first national level survey on public knowledge about antibiotics in Thailand. Most other studies have been conducted in specific groups of the population such as patients, migrants, consumers or at sub-national levels [16, 17]. A few studies conducted in European countries and high-income countries represented regional aggregate and national levels [3, 18–21].

The results of the survey demonstrated that the one-month prevalence of antibiotics use among the Thai population was lower than other studies; compared with the general population in Malaysia (16.5% in the last four weeks) [22]. In the WHO multi-country public awareness survey in 2015, data gathered across 12 countries in all regions showed more than one third of people having taken antibiotics within the past month at 35%. Egypt was the country reporting the highest use of antibiotics in the past month at 54%, while Serbia and Barbados reported only 19% [23]. However, 12.3% of respondents in our study reported that they are not confident whether the medicines they used last month were antibiotics or not, which can affect the findings of low prevalence of antibiotic use.

Sources of antibiotics vary according to the health systems context, financing medicines and regulatory conditions around access to antibiotics. In high-income countries where antibiotics are prescription-only medicines, the source of antibiotics is almost always through physicians’ prescriptions; while in most developing countries, access to antibiotics over the counter and self-medication is common. Thailand achieved universal health coverage in 2002 [24] and all essential medicines are covered in the benefit package; this study confirms that the major sources of antibiotics are healthcare facilities (70.0%) and retail pharmacies dispensed by licensed pharmacists (26.7%). These providers are qualified sources of antibiotics provision, and are likely to increase opportunities to give patients trustworthy information on antibiotics and information about AMR.

The results on indications of antibiotic use were different from Eurobarometer 445 on the top three conditions which were bronchitis (18%), flu (16%) and sore throat (14%) in European Union countries [3]. The three common conditions among Thai adults were flu (27%), fever (19.2%) and sore throat (16.8%). Additionally, several studies showed that flu and common colds were the most common reason for antibiotic use followed by other respiratory symptoms [25, 26], inflammatory conditions [27], urinary tract infections and skin infections [28]. The inappropriate use of antibiotics for the treatment of viral infection such as flu, common colds and other symptoms such as sore throat, cough, fever, headache, diarrhea and inflammation should be closely monitored where the possibility of specific and effective interventions can be introduced.

The design of true and false statements in our study, similar to that of Euro-barometer and WHO, facilitated international comparison. The special Euro-barometer 478 conducted in September 2018 showed that the level of knowledge about antibiotics among Europeans was higher than among Thais. Our findings on the levels of knowledge about antibiotics and AMR were much lower than in European countries; we measured by percentages of wrong answers including “Do not know the answer” to the six statements. To a statement on “Antibiotics can kill virus”, 80.6% of Thais and 57.0% of European respondents provided the wrong answer including “Do not know”. 79.8% of Thais and 34.0% of European respondents gave wrong answers and did not know the answer to a statement on “Antibiotics can cure common colds and flu symptoms”. 70.8% of Thais and 32% of European respondents gave wrong answers and did not know the answer to a statement on “Excessive use of antibiotics can result in side effects such as diarrhea”. 38.2% of Thais and 16% of European respondents gave wrong answers and did not know the answer to a statement on “Only stop antibiotics after completing a full course”. Finally, 36.4% of Thais and 15.0% of European respondents did so to a statement on “Unnecessary or inappropriate use of antibiotics can result in ineffective treatment or resistance” [6].

In Thailand, there is a common misunderstanding that antibiotics can treat inflammatory conditions in humans, so we added the following statement: “Antibiotics are not anti-inflammatory drugs”. From the results, we found that 42.9% gave the correct answer to this statement, which was higher than respondents in Hong Kong; one study showed that 14% of the general population in Hong Kong provided the correct answer to this statement [29]. The general low level of knowledge about antibiotics and AMR was reflected in the figure of 2.6% of Thai respondents who gave correct answers to all six statements, and 13.5% who gave wrong answers to all six statements. This finding presents serious concerns about the low levels of knowledge about antibiotic use and AMR among Thai people.

Our findings about the factors associated with levels of knowledge about antibiotics were similar to other studies; the higher the education levels, the higher level of knowledge about antibiotics [14, 27]. A study in Southwest Alberta [14] showed that people who received information about antibiotics and AMR had 5.3 folds higher levels of knowledge than those who did not receive information. Our results are also confirmed by Oyindamola [14], that people who received information were 1.85-fold more likely to have higher knowledge than those who did not receive information. This emphasizes the importance of providing accurate information on antibiotics and AMR to the general population.

Only 17.8% of Thai respondents had received information about antibiotic use and AMR in the past twelve months, almost half that of those in the European study, where 33% had received information [3]. Similarly, this study shows that low levels of education are negatively associated with receiving information which confirmed a study from Poland [30]. Promoting public knowledge on antibiotics and AMR needs to also focus on disadvantaged groups such as poor and uneducated people.

The common sources of information about antibiotics and AMR in this study were doctors (36.1%), other health professionals (24.8%) and pharmacists (17.7%) while other sources such as the media were uncommon. Sources of information vary across countries due to different contexts and policy interventions. For example, a study in Senegal showed that family or friends are the most common source (58.5%), followed by pharmacy staff (54.5%) and doctors or nurses (25%) whereas the Eurobarometer 445 showed that the most common sources were doctors (32%), television advertisements (27%) and television news (26%) [3, 30]. As health professionals were the main source of information about antibiotics and AMR, policy interventions should mobilize physicians, nurses and pharmacists as effective change agents to deliver correct messages during all clinical encounters on the use of antibiotics in order to strengthen AMR awareness in the population.

To improve the rational use of antibiotics, there should be policies which limit easy access to antibiotics. In Thailand, most antibiotics are classified by the Thai Food and Drug Administration as “dangerous medicines” for which prescriptions are not required at retail pharmacies but can only be dispensed by licensed pharmacists. This facilitates self-medication by the population. Antibiotics in the list of Critically Important Antibiotics [31] should be classified as “specially controlled medicines” which require prescription and cannot be readily available in retail pharmacies. Effective law enforcement to control antimicrobial distribution and inappropriate use are needed.

The WHO Strategic Communication Framework for Effective Communications suggests tailored communication in terms of messages, contents and channels to specific target population [32]. This study contributed to the specific design of content, messages and channels of communication to different target populations and also the development of indicators for measuring progress towards a 20% increase in public knowledge and awareness about AMR in the NSP-AMR.

In the context of rapid expansion and wide spread of internet and social media, the government can maximize use and capitalize their potential for the provision of authentic message and knowledge about proper use of antibiotics and create awareness of the emerging global health threats from AMR, and improve antibiotics stewardship [33]. In the UK, a study suggests that the public that use the Internet as a source of health-related information are more likely to be better informed about, and be more responsible with antibiotics [34]. Despite the high potential of internet and social media, its limitation cannot be under-estimated such as rampant unauthentic message can be misleading. While in parallel the conventional media is accessible for those who cannot access internet media.

In consultation with partners, we developed three sub-indicators to monitor progresses. These are a) percentage of Thai adults who provide correct answers to more than 60% of the true false statements and one question; this demonstrates knowledge of antibiotics; b) mean score of adult population who are aware of the importance of antibiotic uses and AMR using a five Likert scale measurement; and c) percentage of adult population who have received information about AMR and antibiotics. Based on the 2017 HWS results, 23.7% of Thai adults who provided correct answers of more than 60% of knowledge of antibiotic part (sub-indicator a) and 17.8% of Thai adults have received information about AMR and antibiotics (sub-indicator c). Though we did not design to measure sub-indicator b in 2017 survey; it was decided to measure in the next round survey in 2019.

Sub-indicator a is the 2017 baseline knowledge about antibiotics in Thai people for monitoring progress biannually through the AMR module in the HWS. We reviewed the WHO multi-Country AMR public awareness survey as a part of the monitoring and evaluation framework of the AMR global action plan; and added relevant questions for monitoring sub-indicator b in the upcoming 2019 HWS.

A few limitations were experienced in this study. Respondents may have limited understandings about drugs to be able to differentiate antibiotics from other medicines leading to incorrect responses for the use of antibiotics; this affects the findings for the one-month prevalence of antibiotic uses. It is important to note that this is a self-reported survey, and this can lead to a degree of bias with respondents providing the answer they believe is expected by NSO interviewers. This bias is prevented by training of interviewers and a field manual. Also the designs of true and false statements as a factual statement are neutral which reduce such biases; for example “Antibiotics kill viruses” or “Antibiotics are effective against colds and flu”.

Unlike a conventional research, this study had embedded AMR module to the national representative population-based survey. AMR is one of the many modules such as illnesses and injuries, health service uses such as outpatient, inpatient and dental care, health promotion and prevention; and out of pocket payment by households. It is not possible to add more independent parameters such as health literacy. Multiple regression is limited by the existing independent parameters in the HWS.

Despite these challenges, the strengths are the integration of the AMR module in the biannual HWS, which is less costly and that knowledge of antibiotics and AMR awareness can be assessed against various independent parameters.

## Conclusion

This study generated evidence on one-month prevalence of antibiotic use in the Thai population and their knowledge about antibiotics and AMR. Findings from multivariate clearly showed that a few population groups are target for public campaign in order to improve knowledge about proper use of antibiotics; these are individuals with low education, members from poor households, men and younger persons.

Further, results indicated that provisions of public information, knowledge about appropriate use of antibiotics and AMR awareness can be provided through health professionals as they are a major source of antibiotics dispensing and information.

The results from the 2017 HWS serve as a baseline for monitoring the progress of NSP-AMR 2017–2021, which aims to increase public knowledge about antibiotics and AMR awareness by 20% by 2021. Moreover, evidence from this survey contributes to partners’ work in the design of an effective communications strategy to increase levels of knowledge and awareness of antibiotics and AMR among the Thai population.

## Supporting information

S1 File Certificate of exemption from Ethics Committee. (PDF)
