# Supplementary material for: Knowledge and use of antibiotics in Thailand: A 2017 national household survey
Source: PLoS One. 2019 Aug 9;14(8):e0220990. doi: 10.1371/journal.pone.0220990 (PMC6688796; doi:10.1371/journal.pone.0220990)
Supplement: S1 File — (PDF) [file pone.0220990.s001.PDF]

สำนักพัฒนาการคุ้มครองการวิจัยในมนุษย์ (สคม.) สถาบันวิจัยระบบสาธารณสุข
Institute for the Development of Human Research Protections (IHRP),
Health Systems Research Institute (HSRI)

ที่ สคม. 864.1/2561

21 ธันวาคม 2561

เรื่อง ยกเว้นพิจารณาจริยธรรมโครงการวิจัย

เรียน สพญ.สุณิชา ชานวาทิก

สิ่งที่ส่งมาด้วย หนังสือรับรองการยกเว้นพิจารณาจริยธรรมโครงการวิจัย

ตามที่ท่านได้ขอยกเว้นการพิจารณาจริยธรรมการวิจัยในมนุษย์ (Waiver of ethical review) ของโครงการวิจัยเรื่อง "การวิเคราะห์ข้อมูลเรื่องความรู้และความตระหนักเรื่องยาปฏิชีวนะและเชื้อดื้อยาต้านจุลชีพของประชาชนในประเทศไทย พ.ศ.2560" ต่อคณะกรรมการจริยธรรมการวิจัยในมนุษย์ฯ นั้น

ในการนี้ ประธานคณะกรรมการจริยธรรมการวิจัยในมนุษย์ฯ ได้พิจารณาแล้ว มีมติยกเว้นพิจารณาจริยธรรมของโครงการวิจัยดังกล่าว

จึงเรียนมาเพื่อโปรดทราบ

ขอแสดงความนับถือ

(นายแพทย์วิชัย โชควิวัฒน)
ประธานคณะกรรมการจริยธรรมการวิจัยในมนุษย์ ฯ
สถาบันพัฒนาการคุ้มครองการวิจัยในมนุษย์

โทรศัพท์.0-2591-3876, 0-2591-3517, 0-2591-3541 โทรสาร.0-2591-415 website: www.ihrp.or.th
อาคาร 8 ชั้น 7 กรมวิทยาศาสตร์การแพทย์ กระทรวงสาธารณสุข นนทบุรี 11000
Building 8 Floor 7, Department of Medical Science, Ministry of Public Health, Nonthaburi 11000 Thailand

คณะกรรมการจริยธรรมการวิจัยในมนุษย์
ในสถาบันพัฒนาการคุ้มครองการวิจัยในมนุษย์
อาคาร 8 ชั้น 7 ห้อง 702 กรมวิทยาศาสตร์การแพทย์ กระทรวงสาธารณสุข นนทบุรี 11000

เอกสารรับรองการยกเว้นพิจารณาจริยธรรมโครงการวิจัย

คณะกรรมการจริยธรรมการวิจัยในมนุษย์ สถาบันพัฒนาการคุ้มครองการวิจัยในมนุษย์ ดำเนินการให้การรับรองการยกเว้นพิจารณาจริยธรรมโครงการวิจัยตามแนวทางหลักจริยธรรมการวิจัยในคนที่เป็นมาตรฐานสากล ได้แก่ Declaration of Helsinki, CIOMS Guideline

โครงการวิจัย: การวิเคราะห์ข้อมูลเรื่องความรู้และความตระหนักเรื่องยาปฏิชีวนะและเชื้อดื้อยาต้านจุลชีพของประชาชนในประเทศไทย พ.ศ.2560

ผู้วิจัยหลัก: สพญ.สุณิชา ชานวาทิก

หน่วยงานที่รับผิดชอบ: สำนักงานพัฒนานโยบายสุขภาพระหว่างประเทศ

เอกสารรับรอง:

1. โครงร่างการวิจัย 30 November 2018, Version 1.

ลงนาม: ....

(นายแพทย์วิชัย โชควิวัฒน)

ประธานคณะกรรมการจริยธรรมการวิจัยในมนุษย์ฯ

วันที่รับรองการยกเว้นพิจารณาจริยธรรม: 21 ธันวาคม พ.ศ.2561

<u>หมายเหตุ</u> ไม่ต้องทบทวนต่อเนื่อง (การแก้ไขเปลี่ยนแปลง, รายงานความก้าวหน้า, รายงานเมื่อเสร็จสิ้นการวิจัย,อื่นๆ)

Ethics Committee
Institute for the Development of Human Research Protections (IHRP)
Building 8 Floor 7 Room 702 Department of Medical Science Ministry Public Health Nonthaburi Thailand 11000

Certificate of Exemption

The Ethics Committee of Institute for the Development of Human Research Protections (IHRP) has exempted the following study which is to be carried out in compliance with the International guidelines for human research protection as Declaration of Helsinki, CIOMS Guideline.

Title of Project: Analysis of knowledge and awareness of antibiotics and antimicrobial resistance in general population in Thailand 2017

Principal Investigator: Sunicha Chanvatik

Responsible Organization: International Health Policy Program (IHPP)

Document Reviewed:

1. Protocol: 30 November 2018, Version 1.

Signature: ....

(Dr.Vichai Chokevivat)
Chairman

Date of Exemption: 21 December, 2018
Note No Continuing review required
